# Oral lichen planus – retrospective study of 563 Croatian patients

**DOI:** 10.4317/medoral.18940

**Published:** 2014-03-08

**Authors:** Vice Budimir, Ivica Richter, Ana Andabak-Rogulj, Vanja Vučićević-Boras, Vlaho Brailo

**Affiliations:** 1 DMD, Dental Unit Sinj, Sinj, Croatia; 2 DMD, Dental Unit Fužine, Fužine, Croatia; 3 DMD, Department of Oral Medicine, School of Dental Medicine, University of Zagreb, Zagreb, Croatia; 4 Professor of Oral Medicine, Department of Oral Medicine, School of Dental Medicine, University of Zagreb, Zagreb, Croatia; 5 Assistant Professor of Oral Medicine, Department of Oral Medicine, School of Dental Medicine, University of Zagreb, Zagreb, Croatia

## Abstract

Objectives: To investigate the epidemiological and clinical characteristics of oral lichen planus (OLP) in a group of Croatian patients seen between 2006 and 2012. Study Design: A group of 563 patients with a diagnosis of OLP was retrospectively reviewed in our clinic. Data regarding age, gender, medical history, drugs, smoking, alcohol, chief complaint, clinical type, localization, histology, treatment and malignant transformation were registered. Results: Of the 563 patients, 414 were females and 149 were males. The average age at the diagnosis was 58 (range 11-94). The most common site was buccal mucosa (82.4%). Most of our patients did not smoke (72.5%) or consume alcohol (69.6%). Patients reported oral soreness (43.3%), mucosal roughness (7%), xerostomia (3%), gingival bleeding (2%) and altered taste (0.5%) as the chief complaint, while almost half of them were asymptomatic (44.2%). The most common types of OLP were reticular (64.8%) and erosive (22.9%). Plaque-like (5.7%) atrophic/erythemtous (4.3%) and bullous (2.3%) type were also observed. Malignant transformation rate of 0.7% was recorded. Conclusions: OLP mostly affects non-smoking middle-aged women. Buccal mucosa is the most commonly affected site. In almost half of the cases patients are asymptomatic. In spite of the small risk for malignant transformation all patients should be regularly monitored.

** Key words:**Oral lichen planus, malignant transformation, epidemiology, retrospective study.

## Introduction

Oral lichen planus (OLP) is a chronic mucocutaneous autoimmune disorder characterized by an epithelial basal cell lesion that involves a type IV hypersensitivity reaction, which is mostly mediated by the T lymphocyte population (1,2). The prevalence of the disease in the general population is 0.1-4% (3). OLP usually affects middle aged and elderly people (4) with a female/male ratio 2:1 (5).Exact aetiology of OLP still remains unknown. However, it is believed that in most cases it is a multifactorial process which consists of genetic, psychological and infectious factors (2). Intraoraly, buccal mucosa, tongue and gingiva are most commonly involved while other areas like mucosa of the palate and floor of the mouth are rarely affected (1). Clinical presentation can range from asymptomatic white keratotic lesions to painful erosions and ulcerations (6). There are six clinical forms of oral lichen planus: reticular, papular, plaque-like, erosive, atrophic and bullous (5). The most common are reticular and erosive form (1). Histopathological characteristics of OLP are dense subepithelial lymphocytic infiltrate, lymphocitic invasion of epithelium and hydropic degeneration of basal keratinocytes (5).

The treatment of OLP is symptomatic while asymptomatic forms are not treated. Corticosteroids are the most commonly used drugs. Other drugs, like calcineurin inhibitors, azathyoprine, mycophenolate mofetil, retinoids, dapsone and hydroxychloroquine can be used in recalcitrant cases (7).

The most concerning fact about OLP is its potential to develop into oral squamous cell carcinoma (5). Therefore the World Health Organization classified OLP as potentially malignant disorder in 1978 (8). Therefore every patient diagnosed with OLP should be regularly monitored since malignant transformation can occur in all forms of OLP (9). According to the literature frequency of malignant transformation varies from 0 % to 12.5% (2).

The aim of this retrospective study was to evaluate sociodemographic and clinical data of 563 Croatian OLP patients.

## Matherial and Methods

A retrospective chart review of OLP patients treated at the Department of Oral Medicine, School of Dental Medicine, University of Zagreb from 1st January 2006 to 31st December 2011 was performed. Data regarding age, gender, medical history, drugs, smoking and alcohol consumption were registered for 563 patients. Furthermore, clinical data about chief complaint, clinical type of OLP, localization, histology, treatment and malignant transformation were also registered.

The diagnosis of OLP was based on clinical criteria (8). OLP was classified into six clinical types according to the following criteria:

• Reticular – presence of lacelike keratotic lesions on the oral mucosa.

• Papular – presence of small (1 mm) keratotic papules on oral mucosa.

• Plaquelike – presence of plaque-like keratotic lesions on oral mucosa.

• Atrophic/erythematous – presence of areas of thinned/atrophic epithelium within previously defined keratotic lesions.

• Erosive – presence of well-defined erosions within abovementioned lesions.

• Bullous- presence of bullae combined within abovementioned lesions.

Biopsy was performed in atypical cases. Histopathological criteria according to WHO were used (10).

Data were organized in MSExcell® spreadsheets and presented in descriptive manner. Chi square test was used where appropriate. p values lower than 0.05 (*p*<0.05) were considered significant.

## Results

Study population consisted of 414 (73.5%) females and 149 (26.5%) males (ratio F:M=2.8:1). The patient age range was 17-94 years with a mean age of 67.12 ± 23.8 years. Majority of patients did not smoke (433;78.4%) and did not consume alcohol (390;70.9%). Females took significantly more systemic medication than men (72.5% vs. 56.5%; *p*=0.0004) and consumed alcohol in significantly smaller proportion (22.2% vs. 48.6%; *p*=0.000009). Significant differences were also found in the prevalence of systemic diseases (*p*=0.025) ( Table 1).

**Table 1:**
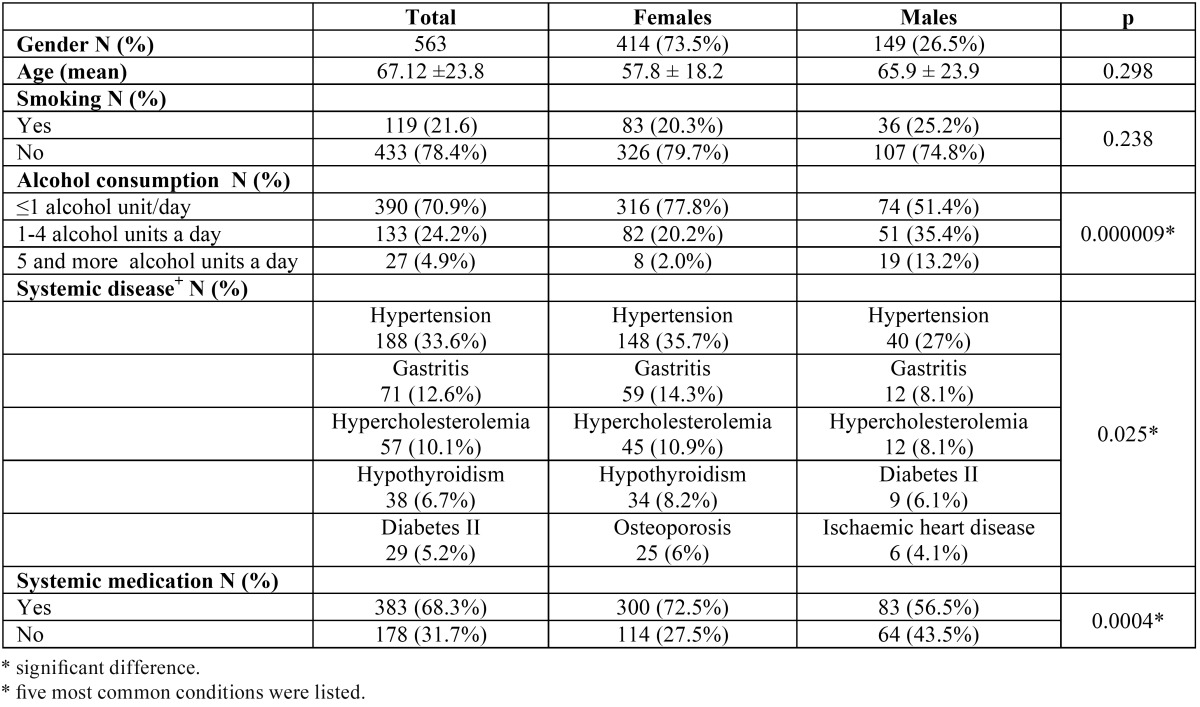
Demographic characteristics of OLP patients.

The most frequent chief complaint in patients was oral soreness (242;43.3% of the patients), followed by mucosal roughness (39;7% of the patients), xerostomia (17;3% of the patients), gingival bleeding (11;2% of the patients) and taste alteration (3, 0.5% of the patients). Nearly half of the patients (247;44.2% of the patients) reported no symptoms. Women reported symptoms in significantly higher proportion than men (59.6% vs. 45.2%; *p*=0.005) ( Table 2).

**Table 2:**
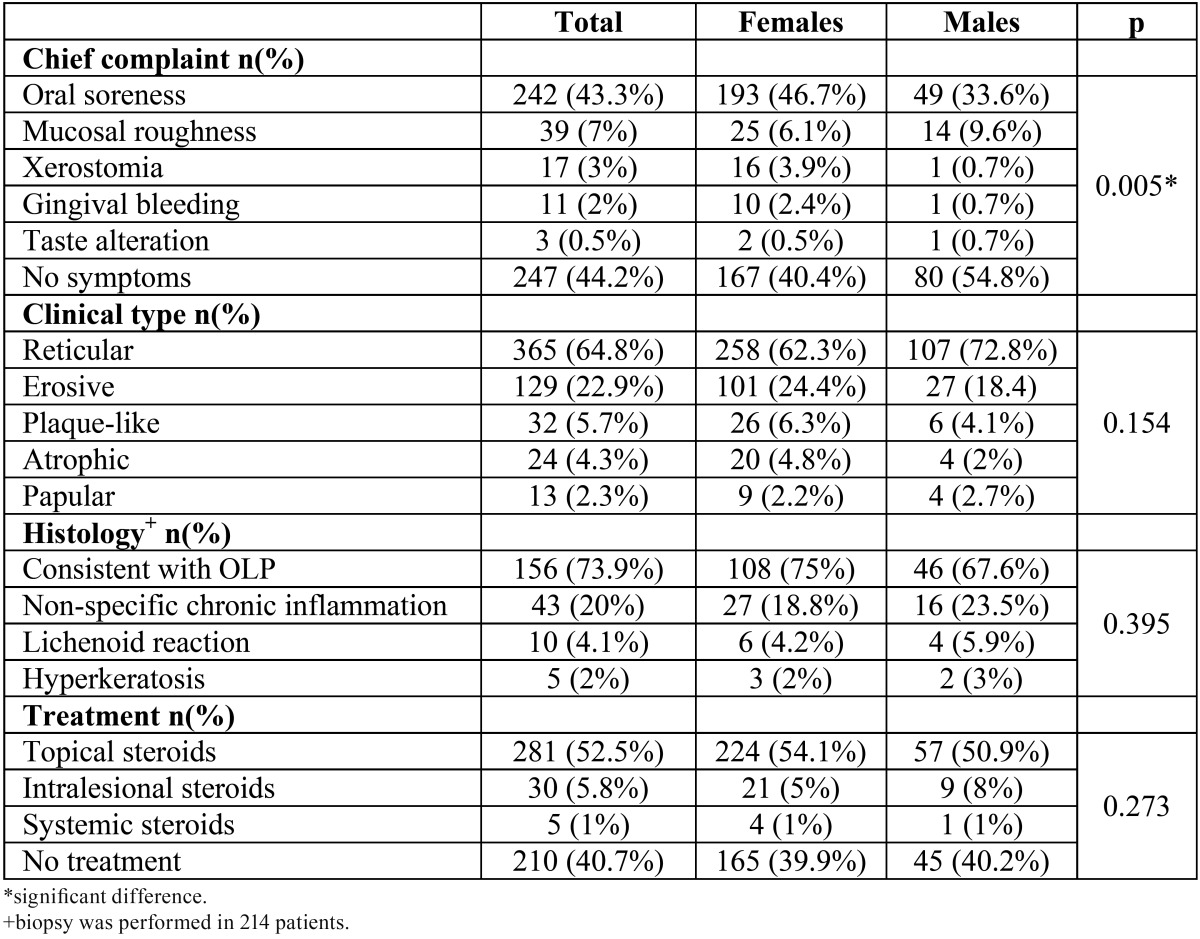
Clinical characteristics of OLP patients.

Reticular type of OLP was most common (365;64.8%) followed by erosive (129;22.9%), plaque-like (32;5.7%) atrophic/erythematous (24;4.3%) and papular type (13;2.3%). No significant differences among females and males were observed ( Table 2).

**Figure 1:**
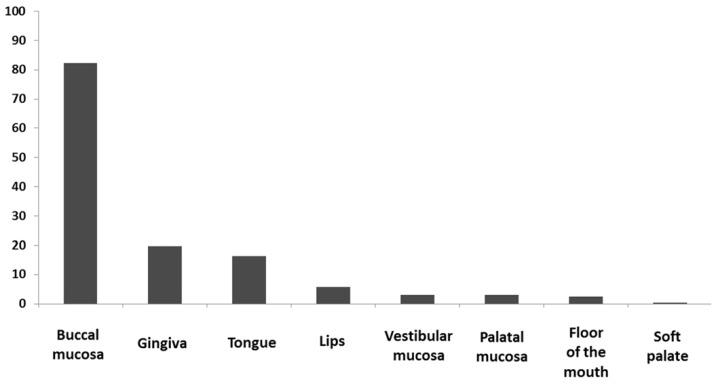
Localisation of OLP lesions (% of patients).

The most affected oral site was buccal mucosa 82.4%, followed by gingiva 19.7% and tongue 16.3%. Distribution of oral lesions is presented in figure 1. No significant differences among females and males were observed.

Biopsy was performed in 214 (38%) patients. Histological diagnosis was consistent with OLP in 156 (73.9%) patients. Other histological diagnoses were non-specific chronic inflammation (43;20%), lichenoid reaction (10;4.1%) and hyperkeratosis (5;2%) ( Table 2).

Treatment was not necessary in 210 (40.7%) patients due to the lack of clinically visible inflammation and lack of symptoms. Topical steroids were used in 281 (52.5%) patients, intralesional steroids in 30 (5.8%) patients and systemic steroids in 5 (1%) patients ( Table 2).

Malignant transformation occurred in 4 (0.7%) female patients (2 with erosive OLP, one with atrophic and one with plaque-like OLP). One patient was active smoker, one was non-smoker and two were ex-smokers (mean time from smoking cessation 2.5 yrs). Mean age of the patient at the time of malignant transformation was 68.5 (SD ± 5.4). Malignant transformation occurred after a mean follow up time of 7.6 years (SD ± 10.7 yrs).

## Discussion

Results of our study are in concordance with similar studies found in literature and confirm that OLP is more frequently found in middle-aged women. Female to male ratio in this study was 2.8:1 which is in concordance with majority of the studies where it varied from 1,6:1 (11) to 3.3:1 (12). However, we found two studies that have shown that both sexes are almost equally affected (13,14).

The age of our patients ranged from 17 to 94 years with the mean age 67.12±23.8 years. These results indicate that our patients might be somewhat older than patients reported in other studies where mean age was reported to be between 50 and 57 (4,11,15,16). Regardless of that difference, majority of our patients were in their 5th or 6th decade where the peak incidence of OLP is reported (4,11,15,16). On the contrary, Pakfetrat *et al.* (6) reported the peak incidence of OLP between ages 30-44 among 490 Iranian patients. Children are rarely affected with OLP. The prevalence of OLP in childhood is reported to be 0.03% (17).

Smoking and alcohol drinking was not common finding among our patients. Four hundred thirty three patients (78.4%) were non-smokers which is similar tothe results reported elsewhere (5,6,12). Great majority (70.9%) of the patients did not drink alcohol which was also reported in other studies (5,6,12). These findings confirm that OLP patients have no increased prevalence of smoking or alcohol abuse compared to the general population (18).

The most common chief complaint in our patients was oral soreness which was reported by 242 (43.3%) patients. Other symptoms like mucosal roughness (39;7%), xerostomia (3%), gingival bleeding (2%) and altered taste (0.5%) were observed in significantly smaller proportion. In nearly half of the patients no symptoms were present (44.2%). Other studies reported similar symptoms in OLP patients and report oral soreness as the most frequent one (4,6). However, the ratio of asymptomatic patients in different studies varied from 16-60% (2,4,6,12).

Buccal mucosa is the most common site of OLP and is reported to be affected in 73%-95.5% of the patients (1,2,4-6). Our results fall within that range with 82.4% patients having bilateral buccal involvement. Other sites that are usually affected are gingiva and tongue. We found tongue involvement in 16.3% of the patients and gingival involvement in 19.7% of our patients. Gingival involvement, on the other hand, was a rare finding (5%) among 518 Chinese patients (5). Sites like palate and floor of the mouth are usually affected in less than 5% of the cases (1,2,4-6).These sites were also rarely affected among our patients (hard palate in 17 (3%) of the patients, floor of the mouth in 14 (2.5%) patients and soft palate in 2 (0.4%) of the patients, respectively).

The diagnosis of OLP can usually be made based only on the clinical findings. However in atypical cases, clinical diagnosis must be confirmed by histopathological report. The differential diagnosis may include cheek chewing/frictional keratosis, lichenoid reactions, leukoplakia, lupus erythematosus, pemphigus, mucuos membrane pemphigoid, erythematous candidiasis and chronic ulcerative stomatitis (7). We performed biopsy only in cases where clinical diagnosis was not straightforward, which was 214 (38%) patients. In the majority of the patients (156;73.9%) the histopathological report was consistent with OLP. The results of other retrospective studies also showed that patients with clinical features of oral lichen planus usually have histopathological features as well (4-6).

Since the aetiology of OLP is still unknown there is no etiological treatment of the disease (19). The aim of the treatment is to relieve the symptoms and minimize the functional impact of the disease. Patients with reticular or papular lesions that don’t pre-sent any symptoms do not need any kind of treatment. On the other hand, erosive lesions are very painful, and in those patients treatment is required. Corticosteroids are usually the first choice for treatment of OLP.

Spontaneous remission of OLP is rare and is reported to vary between 2.47% (11) and 13% (4). Majority of patients continue to have lesions, irrespective of the treatment modality. We found only one patient whose OLP spontaneously remitted.

The most serious complication of OLP is the development of oral cancer, which occurred in 4 (0.7%) of our patients. The rate of malignant transformation of OLP is reported to be 0.07% to 5.8% (2,4,6-9,11,20-22), which places our patient population among ones with lower malignant transformation rates. Malignant transformation is more common among women (5,12,15), even though there are studies that report higher incidence of malignant transformation among men (4). Erosive and atrophic types of OLP carry the greatest risk of malignant transformation even though it can happen in any clinical type. This was the case in our population where two patients who underwent malignant transformation had erosive form of OLP, one had atrophic and one had plaque-like form of OLP, respectively. Smoking and alcohol consumption is not associated with increased risk of malignant transformation of OLP as majority of cases occurs in non-smokers and non-drinkers (5,12,15).

In conclusion, OLP is chronic mucosal disease affecting mainly non-smoking middle-aged women. OLP mostly affects buccal mucosa but all oral regions can be affected. Lesions are symptom-free in nearly half of the cases. Women with erosive/atrophic form of OLP are at greater risk for malignant transformation. This study sheds additional light on epidemiological and clinical features of OLP patients from South Eastern Europe. It also provides evidence of its potentially malignant course emphasizing the need for regular monitoring of all patients.
